# Pericytes, an overlooked player in vascular pathobiology

**DOI:** 10.1016/j.pharmthera.2016.11.008

**Published:** 2017-03

**Authors:** David Ferland-McCollough, Sadie Slater, Jai Richard, Carlotta Reni, Giuseppe Mangialardi

**Affiliations:** Division of Experimental Cardiovascular Medicine, School of Clinical Sciences, Bristol Heart Institute, University of Bristol, United Kingdom

**Keywords:** AGE, Advanced Glycation End-Products, ANG1, angiopoietin-1, ANG2, angiopoietin-2, BBB, blood-brain barrier, BRB, blood-retina barrier, CKD, chronic kidney disease, CNS, blood-retinal barrier, CSC, cancer stem cell, DAN, diabetic autonomous neuropathy, DME, diabetic macular oedema, DN, diabetic nephropathy, DPN, diabetic peripheral neuropathy, DR, diabetic retinopathy, EC, endothelial cells, ECM, extracellular matrix, ED, erectile dysfunction, EGFR, EGF receptor, EMT, epithelial-mesenchymal transition, FGF-9, fibroblastic growth factor 9, GFR, glomerular filtration rate, GSC, glioblastoma CSC, GSI, g-secretase inhibitor, HB-EGF, heparin-binding EGF-like growth factor, HIF, hypoxia inducible factor, HPC, hemangiopericytoma, I/R, ischemia-reperfusion, IL-6, interleukin 6, IL-8, interleukin 8, iPS, induced pluripotent stem cells, MAPK, mitogen-activated protein kinase, MDSC, myeloid-derived suppressor cells, MF-EGF8, milk fat globule epidermal growth factor VIII, MI, myocardial infarction, MMP, matrix metalloproteinases, MSC, mesenchymal stromal cell, NRF2, nuclear factor (erythroid-derived 2)-like 2, NG2, neural/glial antigen 2, NPDR, non-proliferative diabetic retinopathy, Olmfl3, Olfactomedin-like 3, PDL-1, programmed death-ligand 1, PDGFb, platelet-derived growth factor B, PDGFRβ, platelet derived growth factor receptor β, PDR, proliferative diabetic retinopathy, PEDF, Pigment Epithelium-Derived Factor, PKC, protein kinase C, PSC, perivascular stem cell, RAGE, receptors of AGEs, ROS, reactive oxygen species, SDF-1, stromal derived factor 1, SFT, solitary fibrous tumour, SMA, smooth muscle actin, SOD, super oxide dismutase, T1D, type 1 diabetic, T2D, type 2 diabetic, TGF β, transforming growth factor β, TME, tumour microenvironment, Treg, regulatory T cells, UUO, unilateral ureteric obstruction, VEGF, vascular endothelial growth factor, Pericytes, Perivascular stem cells, Diabetic retinopathy, Diabetic nephropathy, Cancer stem cells, Pericyte fibrosis

## Abstract

Pericytes are a heterogeneous population of cells located in the blood vessel wall. They were first identified in the 19th century by Rouget, however their biological role and potential for drug targeting have taken time to be recognised. Isolation of pericytes from several different tissues has allowed a better phenotypic and functional characterization. These findings revealed a tissue-specific, multi-functional group of cells with multilineage potential. Given this emerging evidence, pericytes have acquired specific roles in pathobiological events in vascular diseases. In this review article, we will provide a compelling overview of the main diseases in which pericytes are involved, from well-established mechanisms to the latest findings. Pericyte involvement in diabetes and cancer will be discussed extensively. In the last part of the article we will review therapeutic approaches for these diseases in light of the recently acquired knowledge. To unravel pericyte-related vascular pathobiological events is pivotal not only for more tailored treatments of disease but also to establish pericytes as a therapeutic tool.

## 1 Introduction

Pericytes were first characterized in the 19th century by Rouget as a mural cell population embedded in the basement membrane of venules and capillaries (Bergers and Song, 2005). Due to their perivascular position, Rouget cells were renamed as pericytes by Zimmermann. Initially, pericytes were believed to be involved in vasoconstriction. However, in the last fifty years, the functional properties of pericytes have greatly expanded.

In general, pericytes are involved in the preservation of vascular rheology and homeostasis, including regulation of blood flow, angiogenesis, structural stabilisation of the vasculature, and vascular permeability (Hellstrom et al., 2001, Pallone and Silldorff, 2001, Enge et al., 2002). However, they can also acquire tissue-specific roles. In the central nervous system (CNS) and retina, the ratio of pericytes to endothelial cells (ECs) is 1:1, forming the so-called blood-brain barrier (BBB) and blood-retinal barrier (BRB) (Shepro and Morel, 1993). This pericyte density is essential to form a filter to protect brain and retina cells from potentially toxic blood-derived factors. In the kidney, pericytes are highly specialised cells which account for 30% of the total tissue population and are involved in the regulation of ultrafiltration at the glomerulus (Armulik, Abramsson, and Betsholtz, 2005). In fenestrated endothelium found in organs such as the liver, pericytes – here designated hepatic stellate cells – are important in the remodelling of the extracellular matrix (ECM) (Kitano and Bloomston, 2016). In bone marrow endothelium, pericytes have a role in maintaining the homeostasis of the vascular niche (Corselli et al., 2013a). Emerging evidence demonstrates the involvement of pericytes in immunomodulatory and phagocytic activity (Tu et al., 2011). This plethora of tissue-specific properties demonstrates a high degree of functional plasticity, demanding a more detailed characterization of pericytes.

### 1.1 Pericyte reworked: the perivascular cell world

Due to the heterogeneity of the pericyte population, there is an intense debate as to the definition of pericyte phenotypic characteristics, as well as their potential origin. The common characteristics shared among pericytes are their perivascular position, as determined by electron microscopy, and the ubiquitous expression of platelet-derived growth factor receptor β (PDGFRβ) and CD146 (Crisan et al., 2008). However, pericytes also express divergent markers based upon their location; arterial pericytes express neural/glial antigen 2 (NG2) and α smooth muscle actin (α-SMA), while those localised to the capillary lack these markers (Crisan, Corselli, Chen, and Peault, 2012).

In recent years, a pericyte-like population has been characterized in the tunica adventitia of vessels in different tissues (Campagnolo et al., 2010, Corselli et al., 2012, Avolio et al., 2015, Kramann et al., 2016). These populations share part of their antigenic profile with conventionally-defined pericytes, including NG2/CSPG4 and PDGFRβ, but do not express CD146. Notably, they can express CD34, a typical marker of hematopoietic progenitor cells (Traktuev et al., 2008). To what extent this adventitial population should be considered related to microvascular pericytes remains uncertain. Both pericytes and adventitial cells have been demonstrated to commit to different mature mesoderm-like lineages (such as osteoblast, chondrocyte, and adipocyte), (Paquet-Fifield et al., 2009, Dellavalle et al., 2011, Katare et al., 2011, Park et al., 2011, Paul et al., 2012), thus suggesting a multipotent capability similar to mesenchymal stromal cells (MSCs) (Bara, Richards, Alini, and Stoddart, 2014). Accordingly, the whole perivascular cell population has been suggested to be the *in situ* equivalent of MSCs, while MSCs have been proposed as the *in vitro* counterpart of pericytes (Crisan et al., 2008). However, the International Society for Cell Therapy states that MSCs represent a heterogeneous cell population with different differentiation capabilities (Dominici et al., 2006) and Blocki et al. have recently demonstrated that not all MSCs can differentiate into pericyte-like cells (Blocki et al., 2013). In this context, Birbrair et al. have characterized *in vitro* two distinct pericyte populations: type-1 pericytes, able to generate adipocyte and fibroblasts but not neural cells, and type-2, characterized by neurogenic and myogenic potential (Birbrair et al., 2014). Moreover, *in situ* characterization of MSCs demonstrates that those expressing CD146 resemble pericytes in their localisation (peri-endothelial) and their angiocrine activity (Corselli et al., 2013b). Therefore, pericytes and adventitial progenitor cells can be grouped as perivascular stem cells (PSCs) according to a) their localisation and b) their multipotency (James et al., 2012, Askarinam et al., 2013, Chung et al., 2015).

The theory that pericytes are merely supportive perivascular cells can now be considered obsolete. According to the characteristics described above, these cells should be considered as heterogeneous, tissue-specific, and multipotent populations. The aim of this review, therefore, is to provide a general description of the perivascular cell population, highlight their recently described roles in the development of different pathophysiological processes and discuss how this is being exploited in pericyte-targeted therapies.

## 2 Diabetes

### 2.1 Diabetic retinopathy

Diabetic retinopathy (DR) is a major complication of diabetes. In the UK, diabetes is the leading cause of blindness. Patients suffering from diabetes have a 10 to 20 times increased risk of developing blindness compared to non-diabetic individuals. DR symptoms include blurred vision, the appearance of dark spots, the perception of “floaters” in the field of vision, eye pain, double vision, reduction in low-light perception, sudden vision loss, and complete blindness. Risk factors for DR include high blood pressure, hyperglycemia, hyper- or dyslipidemia, ethnicity, as well as the type of diabetes. Within the first 2 decades after disease onset, nearly all type 1 diabetic (T1D) patients will develop retinopathy, compared with 60% of type 2 diabetic (T2D) patients (Fong et al., 2004).

**Fig. 1:**
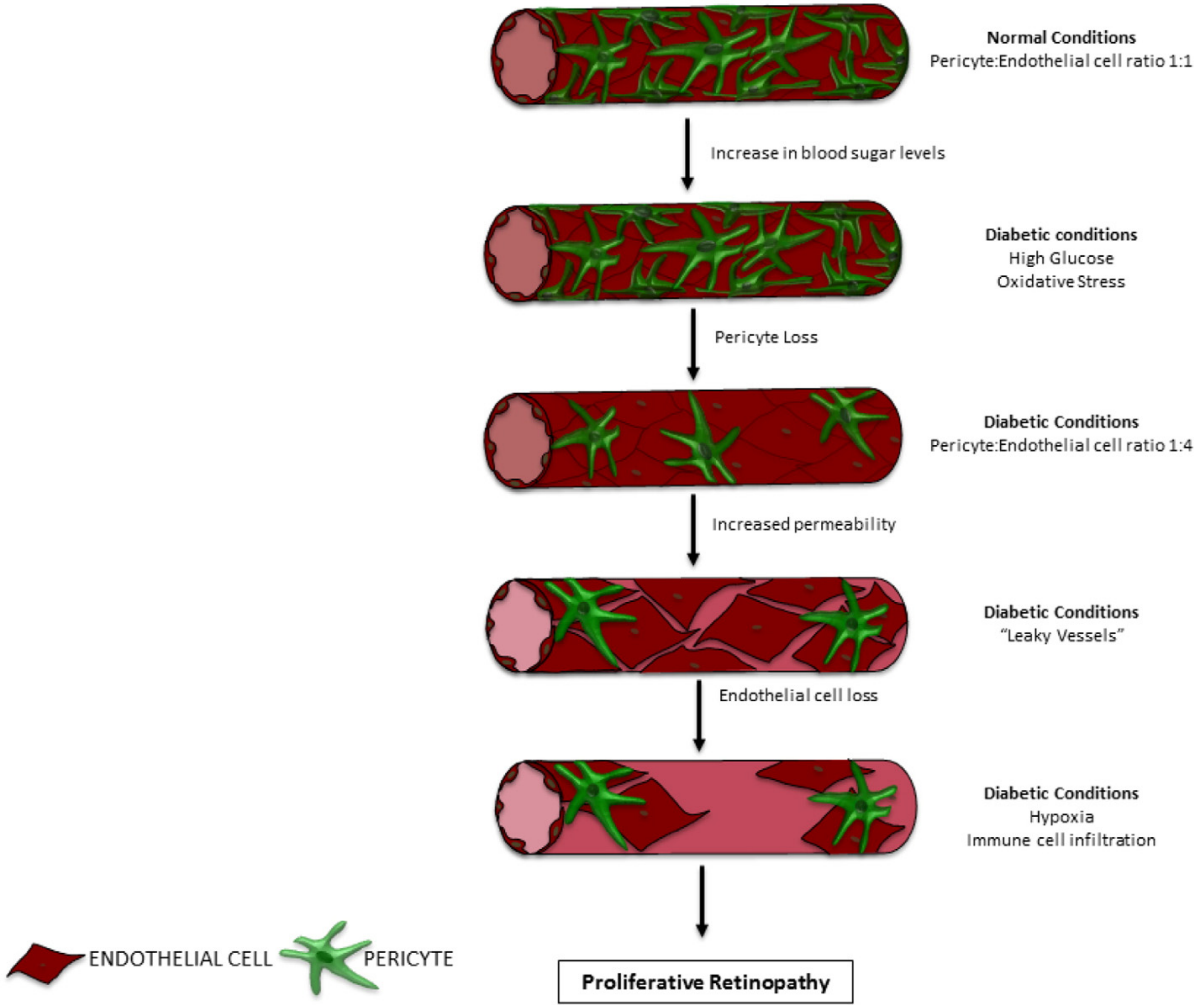
Progression of diabetic non-proliferative retinopathy. Progression of early-stage non-proliferative retinopathy to proliferative retinopathy. Elevated glucose levels will cause apoptotic cell death of pericytes. This will lead to a more permeable blood vessels and subsequent endothelial cells which will increase leakiness of vessels even more. Loss of pericytes and endothelial will increase fluid leakage in the retina as well as immune cell infiltration. These intra-ocular vascular changes will then contribute to the development of proliferative retinopathy.

DR can be described as a microvascular disease that eventually affects all cell types in the retina. The pathology develops in two different stages: non-proliferative diabetic retinopathy (NPDR) and proliferative diabetic retinopathy (PDR) (Fig. 1). NPDR is the earliest stage of the disease and can be classified in four forms: mild, moderate, severe and very severe. NPDR patients' retinas exhibit microaneurysms, microhemorrhages, nerve fibre infarcts (also known as cotton wool spots), retinal oedema, and intraretinal vascular abnormalities. On the cellular level, NPDR is characterized by a loss of both pericytes and ECs, causing a decrease in the number of functional blood vessels as well as disruption of the BRB. Advanced PDR is characterized by the proliferation of blood vessels throughout the retina and an increase in basement membrane thickness. Neovascularization and the related dysregulated endothelial sprouting support the development of highly irregular vascular networks leading to the penetration of the choroid and vitreous areas of the eye. Vessel invasion can cause obstruction and even detachment of the retina, leading to blindness. DR can also lead to diabetic macular oedema (DME), which is characterized by the swelling of the maculae caused by leaking of fluids from the aberrant blood vessels. The macular region of the retina is rich in colour sensing cones, therefore microvascular leakage in this region leads to disruption of these light-sensing cells and a decrease in visual perception.

Pericytes are an integral component of both the retina and its microvasculature. The retina is the highest energy-consuming structure in the human body, requiring even more resources than the brain (Schmidt et al., 2003). The importance of pericytes to the retinal microvasculature is highlighted by the fact that the ratio of retinal pericytes to ECs is 1:1, and that they cover 85% of the microvasculature area (Shepro and Morel, 1993). The pericyte-EC interaction in the retina is a primordial part of the BRB, which tightly regulates the transport of nutrients, oxygen, growth and regulatory factors as well as immune cells from the periphery into the neuronal environment of the retina. Disruption of endothelial-pericyte interactions leads to “leaking” of the vasculature into various regions of the retina and facilitates access of immune and inflammatory cells into the retinal environment.

One of the earliest hallmarks of NPDR is the loss of pericytes. This cell death causes regression of the microvasculature leading to leaking of fluids, leukocyte adhesion to the vasculature and hypoxia in the damaged area. The diabetic microenvironment seems particularly detrimental to pericyte survival; during the early stages of retinopathy, the pericyte to EC ratio decreases from 1:1 to 1:4 (Robison, Kador, and Kinoshita, 1985).

The high glucose microenvironment in diabetes can directly induce apoptosis of retinal pericytes (Haribalaganesh, Sheikpranbabu, Elayappan, Venkataraman, and Gurunathan, 2009). High glucose-induced cell death is due to an increase in production of reactive oxygen species (ROS). Increased ROS can disrupt the mitochondrial membrane, leading to the release of cytochrome *C* and the activation of the caspase-3 dependent apoptotic cascade. It has been suggested that hyperglycemia-related complications are due to the increased production of superoxide through an increased output of the mitochondrial electron transport chain (Nishikawa et al., 2000). More recently, it has been demonstrated that only the inhibition of NADPH oxidase is able to abrogate hyperglycaemic-related events such as ROS production and *in vitro* pericyte apoptosis (Mustapha, Tarr, Kohner, and Chibber, 2010).

Another mechanism by which high glucose-induced oxidative stress can cause pericyte loss in DR is through the generation of Advanced Glycation End-Products (AGEs). AGEs are formed when reducing sugars such as glucose react in a non-enzymatic manner with nucleic acids, proteins, and lipids. Intracellularly, these reactions can result in DNA and RNA damage, protein crosslinking, and lipid peroxidation, leading to multiple cytotoxic effects, reviewed in (Singh, Barden, Mori, and Beilin, 2001). *In vitro*, hyperglycemia and AGE generation also induce free-radical formation, which leads to oxidative stress and apoptosis of retinal pericytes (Assero, Lupo, Anfuso, Ragusa, and Alberghina, 2001).

Extracellular AGEs can also affect pericytes. Receptors of AGEs (RAGE) are found on most cell surfaces and can exert multiple effects depending on the cell type. In the case of the retinal microvasculature, AGE-RAGE binding increases EC proliferation through an autocrine mechanism involving the mitogen-activated protein kinase (MAPK) ERK pathway and hypoxia-inducible factor 1α (HIF1α) activation leading to the expression of vascular endothelial growth factor (VEGF.) AGE-RAGE interaction also induces VEGF production by retinal pericytes (Yamagishi et al., 2002b). This increase in VEGF production is one of the major contributors to the development of PDR and mediates progression of the disease. Although AGE-RAGE interaction increases retinal EC proliferation, in pericytes it induces apoptosis and is thought to be a major contributor to the pericyte loss observed in early NPDR. *In vitro*, treatment of retinal pericytes with extracellular AGEs causes apoptosis in a dose-dependent manner (Yamagishi et al., 2002b). *In vivo*, streptozotocin-induced T1D RAGE^−/−^ mice showed decreased vascular permeability and retinal acellular capillary formation (McVicar et al., 2015). Contrary to expectations, diabetic RAGE^−/−^ mice did not exhibit a decrease in pericyte loss, suggesting that alternative pericyte pro-apoptotic factors are at work in DR.

The damaging effects of ROS and AGEs are exacerbated by the downregulation of expression and/or activity of various anti-oxidant proteins in the diabetic retina. Nuclear factor (erythroid-derived 2)-like 2 (NRF2) is responsible for the transcription of a large number of detoxifying enzymes, *e*.*g*. heme oxygenase and glutathione, and the knockout of NRF2 in diabetic mice causes exacerbation of DR (Xu et al., 2014). It has been reported that NRF2 nuclear translocation and transcriptional activity is suppressed in DR both *in vivo* and *in vitro* (Zhong, Mishra, and Kowluru, 2013). Sulforaphane is a known activator of NRF2 both *in vivo* and *in vitro* (Jimenez-Osorio, Gonzalez-Reyes, and Pedraza-Chaverri, 2015). It has been shown that sulforaphane treatment of bovine retinal pericytes *in vitro* decreases ROS production and AGE formation, as well as RAGE mRNA and protein expression (Maeda, Matsui, Ojima, Takeuchi, and Yamagishi, 2014).

The levels in the aqueous humour of another player in the ROS-detoxification pathway, Pigment Epithelium-Derived Factor (PEDF), have been positively correlated with the anti-oxidant status of the retina (Yoshida et al., 2007). PEDF levels are decreased in the vitreous of diabetic patients with retinopathy compared to healthy subjects (Boehm et al., 2003b), and are a strong predictor of progression of NPDR (Boehm et al., 2003a). PEDF has multiple roles in the microvasculature. Increased production of PEDF has been shown to reduce ROS-induced apoptosis by stabilising the mitochondria *via* activation of the PI3K/AKT survival pathway (He et al., 2014a). PEDF can also act directly on ROS production. In cultured retinal pericytes, PEDF treatment reduced the production of ROS induced by oxidized low-density lipoproteins (ox-LDL). PEDF exerts its effects through the blockade of the NF-kappaB pathway and the transcriptional upregulation of superoxide dismutase (SOD) 1 (Zhang et al., 2008). Similarly, PEDF can abrogate the negative effects of AGEs through both its anti-oxidant effects and by directly acting on survival pathways. PEDF treatment increases the expression of the detoxifying enzymes SOD and glutathione reductase which impairs the generation of ROS by AGEs (Maeda et al., 2014). PEDF can also induce cell survival by activation of the Src kinase pathway (Sheikpranbabu, Haribalaganesh, and Gurunathan, 2011), as well as by increasing the levels of the anti-apoptotic protein Bcl-2 (Yamagishi, Inagaki, Amano, et al., 2002a). Furthermore, PEDF inversely modulates VEGF expression, by acting as a transcriptional repressor of VEGF in retinal ECs (Zhang, Wang, Gao, Parke, and Ma, 2006).

While high glucose and AGEs are factors affecting the whole retinal environment, signals from specific cells within the retina can also contribute to pericyte loss. The angiopoietin/Tie2 pathway is a central pathway in pericyte-EC crosstalk and regulation. angiopoietin-1 (Ang1) is expressed by pericytes while the antagonist, angiopoietin-2 (Ang2) and its receptor Tie2, are expressed by ECs. In basal conditions, Ang1 secreted by pericytes stimulates ECs to produce transforming growth factor β (TGF-β) and platelet-derived growth factor B (PDGFb). These factors, in turn, stabilise pericyte-EC interactions (Armulik et al., 2005), while antagonism of this pathway leads to destabilisation of pericyte-EC interactions. Ang2 production by ECs is specifically upregulated during angiogenesis (Gale et al., 2002). It has been shown that Ang2 protein levels in the diabetic retina can be dramatically increased compared to non-diabetic controls and that injection of recombinant Ang2 in the retina causes dramatic pericyte loss (Park et al., 2014). Park et al. have recently shown that concomitant hyperglycemia and Ang2 stimulation reduce the p53-dependant apoptosis of retinal pericytes. Interestingly, Ang2 modulates this process not by interacting with Tie2, but with the novel α3β1 integrin (Park et al., 2014).

### 2.2 Diabetic nephropathy

Diabetic nephropathy (DN) resulting from the poor control of circulating glucose levels is a leading cause of chronic kidney disease (CKD) in the Western world and is responsible for 30–40% of all end-stage renal disease in the UK (Kidney Research UK, 2015). It is characterized by persistent high protein levels in the urine (proteinuria; > 300 mg/24 h), progressive decline in the glomerular filtration rate (GFR) and increased arterial blood pressure. Renal pericytes are particularly affected by high glucose, leading to the pathology of DN. Current therapies for the treatment of DN include strict control of blood glucose by diet and/or administration of insulin, and control of blood pressure with antihypertensive agents, such as angiotensin-converting enzyme inhibitors or angiotensin II receptor blockers. While these treatments can slow the progression of DN, they cannot prevent or reverse it. Current clinical trials are focusing on targeting the specific molecular pathways involved in renal oxidative stress and fibrosis, which has recently been reviewed in detail by Khan and Quaggin (2015).

Renal pericytes are found around the peritubular capillaries that surround the nephron, where the ratio of pericytes to ECs is 1:2.5 (Armulik et al., 2005). Furthermore, a specialised form of pericyte, known as mesangial cells, are located in the glomerulus, the filtration unit of the kidney. There is some debate as to whether glomerular podocytes could also be classed as a specialised type of pericyte. During development, the metanephric mesenchyme differentiates into nephrons, resulting in both stromal and epithelial lineages. In 1996, Hatini, Huh, Herzlinger, Soares, and Lai (1996), described that stromal cells, which become pericytes, vascular smooth muscle cells (VSMCs), and mesangial cells, express the transcription factor FoxD1. It has subsequently been recognised that podocytes also highly express FoxD1, suggesting they descend from the FoxD1 lineage (Brunskill, Georgas, Rumballe, Little, and Potter, 2011). Podocytes share many characteristics of pericytes and therefore have been included in this review as pericyte-like cells. Peritubular pericytes, mesangial cells, and podocytes are all found wrapped around capillaries, embedded in the matrix they secrete (Diaz-Flores et al., 2009) and form close contacts with the underlying ECs (Kida and Duffield, 2011). They all have roles in stabilising the vascular networks (von Tell, Armulik, and Betsholtz, 2006), and modulating vascular tone (Saleem et al., 2002, Crawford et al., 2012) and vascular permeability (Satchell et al., 2002).

High glucose causes migration of peritubular pericytes away from the capillary into the interstitial space. The capillary becomes destabilised, resulting in microvascular rarefaction. This, in turn, can lead to tissue hypoxia. The migrating peritubular pericytes are thought to transform into myofibroblasts, as these cells are not seen in the healthy kidney. Lin et al. proposed pericytes as the source of myofibroblasts. They labelled collagen type I producing cells (peritubular pericytes) in the kidney with green fluorescent protein and showed that these cells initially expressed typical pericyte markers (PDGFR-β, CD44, CD90 NG2 and α-SMA), but expression decreased during kidney maturation (Lin, Kisseleva, Brenner, and Duffield, 2008). Kidney injury upregulated expression of the above genes in pericytes, in particular α-SMA. It also upregulated other genes associated with the myofibroblast phenotype, along with an increased production of matrix proteins, in particular collagen type I. The pericytes migrated away from capillaries into the interstitial space, where deposition of excess matrix led to tubulointerstitial fibrosis and loss of organ function (Lin et al., 2008). It is worth noting that renal fibrosis is not only seen in response to DN but also in other types of kidney injury, such as unilateral ureteral ligation and ischemia-reperfusion injury.

In the glomerulus, where mesangial cells constitute 30% of all glomerular cells (Schlondorff, 1987), DN is characterized by mesangial cell hypertrophy, proliferation, and an increase in production of mesangial matrix (*e*.*g*.: type IV collagen, laminin and fibronectin) (Stratman, Malotte, Mahan, Davis, and Davis, 2009). Cellular hypertrophy is thought to result from high glucose-induced expression of a number of growth factors, including Ang-2 and TGF-β, which causes protein kinase C (PKC) activation (Li et al., 2007). Brosius et al. also suggest the involvement of mTOR in activating pro-growth and antiapoptotic pathways (Brosius, Khoury, Buller, and Chen, 2010). Meanwhile, Liu et al. (2012a) have shown that increased activity of mTOR, *via* downregulation of Cx43, leads to mesangial hypertrophy in response to high glucose.

Many of the symptoms of DN can be related to an increase in matrix production by renal pericytes (both peritubular and mesangial cells) in response to oxidative stress caused by high glucose. For a comprehensive review of oxidative stress signaling in DN refer to this review by Manda, Checherita, Comanescu, and Hinescu (2015). Briefly, high glucose causes the formation of AGE, which in turn increases ROS production, leading to activation of a number of signaling pathways, *via* PKC (Coughlan et al., 2009). High glucose can directly activate PKC or indirectly through the AGE/ROS axis. This causes activation of MAPK signaling pathway resulting in increased expression of matrix proteins (Lander et al., 1997), and also inhibition of expression of genes involved in matrix degradation, such as matrix metalloproteinases (MMPs) (Fiorentino et al., 2013). Furthermore, MAPK and extracellular ROS can upregulate TGF-β expression in peritubular pericytes, podocytes and mesangial cells (Iglesias-de la Cruz et al., 2002, O'Donovan et al., 2012). TGF-β is thought to be a major pro-fibrotic factor in DN, causing increased expression of both collagen type IV and fibronectin (Ziyadeh et al., 2000, Iglesias-de la Cruz et al., 2002), while inhibiting matrix degradation. The increased deposition of matrix by peritubular pericytes causes tubulointerstitial fibrosis, as described above, and may occlude the lumen of the peritubular capillaries and glomerulus leading to an increase in blood pressure. Furthermore, in the case of mesangial cell matrix expansion, it may affect the GFR by causing glomerular scarring (glomerular sclerosis) and reducing the surface area for glomerular filtration.

High glucose causes changes in podocyte morphology by altering the actin cytoskeleton (Lv et al., 2016) and podocyte loss by the detachment of podocytes from the basement membrane (Dessapt et al., 2009) or by apoptosis (Susztak, Raff, Schiffer, and Bottinger, 2006). Each of these results in increased proteinuria and decreased GFR, because podocytes are terminally differentiated and therefore unable to replicate (Shankland and Al'Douahji, 1999). Mechanisms for glucose-mediated podocyte apoptosis are thought to be mediated through induction of ROS by NADPH oxidase and AGE (Eid et al., 2009) (as discussed above), leading to increased intracellular calcium and apoptosis (Liu et al., 2013). There are also suggestions that TGF-β can reduce podocyte attachment to the glomerular basement membrane (GBM) by downregulating expression of integrins, in particular α3β1 integrin (Dessapt et al., 2009). ROS over-activation may also cause the remaining podocytes to increase production of the basement membrane, causing it to thicken. Interestingly, podocytes express the insulin receptor (Coward et al., 2005) and podocyte-specific deletion of the insulin receptor results in proteinuria, increased glomerular matrix, thickened GBM, and changes in podocyte morphology and effacement (Welsh et al., 2010). This suggests some of the podocyte-related changes seen in DN could be as a result of impaired podocyte insulin signaling.

Similar to pericytes of the retina, podocytes produce VEGF-A. However, here it is primarily involved in regulating capillary permeability (Eremina et al., 2003), rather than neovascularisation. It does this by signaling through VEGFR-2, which is mainly expressed by glomerular ECs. In DN, there is evidence that either too much or too little VEGF-A can be pathological. Overexpression is generally seen at the start of the disease, causing GBM thickening, mesangial expansion, podocyte effacement, detachment, and increased proteinuria (Veron et al., 2010). As the disease progresses, VEGF-A expression decreases to below the normal range (possibly due to the loss of podocytes) and results in increased proteinuria (Baelde et al., 2007). Work by Jin et al. (2012) has described how the deletion of the soluble form of the VEGF receptor (sFlt1) from podocytes caused reorganisation of the podocyte actin cytoskeleton and proteinuria. This further demonstrates the importance of VEGF and its receptors in pericyte biology. This effect was not just localised to renal pericytes. When sFlt1 was silenced in the lung, trachea or retinal pericytes, vascular defects were observed, underlining the importance of pericytes in the maintenance of vascular structure in the microcirculation. Studies by Lin et al. (2011) have demonstrated the importance of cross-talk between ECs and pericytes to maintain the vascular structure. Blocking VEGFR-2 on ECs enhanced pericyte detachment after injury and increased fibrosis. However, blockade of PDGFR-β production by pericytes in response to injury prevented pericyte detachment and reduced fibrosis. These findings suggest modulation of growth factor cross-talk between the cells may offer a novel therapeutic approach for kidney injury and other pathologies relating to microvascular defects.

### 2.3 Diabetic neuropathy

Diabetic neuropathies are a heterogeneous group of disorders that include diabetic peripheral neuropathies (DPN) and diabetic autonomic neuropathy (DAN). These result from metabolic injuries causing diffuse and widespread damage to peripheral and autonomic nerves (Vinik and Erbas, 2013).

Previous studies have shown DPN to be associated with lower limb pain, neural desensitisation, foot/leg abscesses, potentially resulting in the eventual amputation of lower limbs. Similarly, DAN has been shown to induce symptoms in the parasympathetic and sympathetic systems which can result in cardiac anomalies such as myocardial ischemia and sudden death (Pop-Busui, Sima, and Stevens, 2006). Moreover, both DAN and DPN patients exhibit functional and structural impairments in peripheral nerves, caused by oxidative stress, AGE accumulation, glucose toxicity and hypoxia, all eventually leading to neuronal apoptosis. The hypoxic conditions observed in diabetic neuropathy are directly related to microangiopathies of the endoneurial capillaries. Nerve biopsies have shown a decrease in both pericytes and ECs in endoneurial capillaries, (Yasuda and Dyck, 1987). Arguably, the loss of pericytes leads to loss and disorganisation of ECs, resulting in decreased perfusion of peripheral nerves and consequent hypoxia.

Additionally, previous studies have shown that under anoxic conditions electrophysiological parameters of nerve conduction undergo a decrease in both velocity and amplitude, which relates to the loss of feeling in the lower extremities (Punsoni, Drexler, Palaia, Stevenson, and Stecker, 2015). These changes in conduction are associated with structural changes including demyelinated axons and a decreased nerve fibre density, exacerbated by hyperglycemia (Punsoni et al., 2015). Oxygen starvation of nerve fibres leads to oxidative stress due to a shift towards anaerobic energy metabolism. Consequent ROS production leads to the activation of apoptotic pathways resulting in Schwann cell death and therefore disrupted neural conduction (Ma et al., 2013).

### 2.4 Diabetic erectile dysfunction

One of the most common but least understood complications arising from diabetes is erectile dysfunction (ED). Diabetic patients have a three times greater risk of developing ED (McKendry et al., 1983), while also being the most resistant to phosphodiesterase-5 inhibitor treatment (Martinez Jabaloyas, Gil Salom, Pastor Hernandez, Villamon Fort, and Garcia Sisamon, 2002). Classic anti-ED drugs such as sildenafil target the nitric oxide synthesis pathway leading to the release of NO from ECs, which causes VSMC relaxation. The pathobiology of diabetic ED does not appear to be due to aberrant NO production, but instead to endothelial dysfunction resulting in leaky endothelium and deregulation of VSMC contractility (Castela and Costa, 2016). There are very few studies concerning the role of pericytes in erectile dysfunction. It has been shown that both in humans and mice, penile pericytes are principally located in the subtunical area of the *corpus cavernosum* in the periphery of the erectile tissue (Yin et al., 2015). In STZ-induced diabetic mice, a significant decrease in penile pericyte number was observed compared to the control group. This phenomenon was associated with an increase in *corpus cavernosum* sinusoidal permeability. Notably, an increase in ox-LDL extravasation has been observed in the *corpus cavernosum* of diabetic mice (Batbold et al., 2016). Ox-LDL has been linked to inflammation and fibrosis which could impair erectile tissue expandability.

## 3 Involvement of pericytes in ischemic pathophysiology

Ischemia is caused by inadequate arterial blood flow to a specific organ, which leads to multiple degenerative cellular phenomena, namely fibrosis, necrosis, and apoptosis. In extreme cases, these cellular phenomena are translated to organ function disruption and organ failure. Pericytes have an important role in the etiology of ischemic organ failure.

In myocardial ischemia, pericytes have been shown to be involved in fibrosis and scar formation. Using a cardiac ischemia-reperfusion (I-R) injury mouse model it was demonstrated that ischemia results in shortening of pericyte extension processes and elevated expression of p75NTR. Low expression of p75NTR in the microvasculature has been associated with smaller infarct size in animal models. An increased expression in cardiac microvascular pericytes could lead to cardiac fibrosis (Siao et al., 2012). It was also shown that type-1 pericytes were recruited to scar tissue after myocardial infarction (MI) but did not contribute to collagen type 1 production leading to fibrosis (Birbrair et al., 2014).

Fate-tracing experiments in the AT2-induced hypertensive heart disease mouse model and the AAC injury model showed that Gli1^+^ cells differentiated into α-SMA^+^ myofibroblasts in the perivascular and interstitial spaces. These Gli1^+^ cells were MSC-like perivascular cells that express MSC markers, possess trilineage potential and include a small fraction of pericyte-like PDGFRB^+^ cells. Further analysis revealed that approximately 60% of Gli1^+^ cells had the propensity to differentiate into cardiac myofibroblasts (Kramann et al., 2015).

Interestingly, pericytes seem to be a major contributor to the production of myofibroblastic cells and scar formation in the ischemic kidney. Acute or chronic kidney injury/disease is characterized by the formation of fibrotic tissue in the interstitial spaces in the kidney. There have been contradicting views about the origin of myofibroblasts involved in scar tissue formation during tissue repair. Early *in vivo* studies have shown that myofibroblasts were derived from multiple cell types but not pericytes. Studies performed by genetic labeling of cells in mouse models demonstrated that the production of myofibroblasts during kidney injury derived from 50% resident fibroblasts, 35% bone marrow cells, 10% tubular epithelial cells and 5% ECs (Hall et al., 2014). Fibrosis is the result of cell proliferation, differentiation, and epithelial and endothelial to mesenchymal transition. However, further lineage analysis of FoxD1 (LeBleu et al., 2013) and coll1a1 (Humphreys et al., 2010) reporter mice with unilateral ureteric obstruction (UUO) and I/R injury have demonstrated that pericytes/perivascular cells are the major source of myofibroblasts resulting in scar tissue formation (Lin et al., 2008).

In cerebral ischemia, the involvement of pericytes in the pathophysiology seems less clear. A mouse model of stroke showed that Nox4 was upregulated in cells expressing PDGFRβ, a pericyte marker. Upregulation of Nox4 in pericytes resulted in increased MMP-9 production, bringing about the breakdown of the BBB (Birbrair et al., 2014). It has also been shown that during ischemia neurones secrete signals to pericytes lining the cerebral capillaries. These signals cause the constriction of vessels followed by their rapid death (Hill et al., 2015). This claim is currently being challenged, as *in vivo* imaging of double transgenic mice expressing mCherry driven by the α-SMA promoter and endothelial GFP driven by the Tie2 promoter (SMA-mCherry:Tie2-GFP) showed that capillary pericytes lack α-SMA and are non-contractile *in vivo*. They are distinctly different from arteriolar VSMCs that are contractile in nature. This was confirmed by *in vivo* optogenetic stimulation of cells lining the vessels. Activation of VSMCs covering arterioles caused vessel constriction and reduced cerebral blood flow compared to stimulation of pericytes in capillaries where no vessel constriction occurred. This study appears to eliminate pericyte involvement in the early stages of cerebral ischemia (Nishimura et al., 2015).

## 4 Pericytes and tumours

Originally, pericytes were believed to contribute to tumour development by participating in angiogenesis. In recent years, however, new evidence supports the concept of pericytes as one of the main regulators of the tumour microenvironment (TME) through angiogenesis, metastasis, and participation in the cancer stem cells (CSCs) pool.

### 4.1 Pericyte tumours

In 2013 the WHO revised the criteria for the classification of soft and bone tissue tumours. Hemangiopericytoma (HPC) is no longer classified as a pericyte-derived tumour because it is now known to derive from a variety of different cell types (Jo and Fletcher, 2014). Spindle cell lesions known as HPC are now considered as a variety of solitary fibrous tumour (SFT), unrelated to pericytes (Schweizer et al., 2013). Currently, only glomus tumour and myopericytoma are considered as true pericyte-derived tumours. Both tumours share the tendency to grow in a perivascular position and have evidence of myoid differentiation (Jo and Fletcher, 2014). Glomus tumour is a rare neoplasm arising from the thermoregulatory glomus body, with an even rarer malignant variant. It shows a perivascular growth pattern and usually expresses pericyte markers such as vimentin and α-SMA (but rarely desmin), and numerous superficial pinocytotic vesicles (Mravic et al., 2015). Similarly, myopericytoma is characterized by α-SMA, occasionally desmin and CD34, but is negative for protein S100 and cytokeratin (Terada, 2010, Terada, 2012). Both tumours demonstrate positivity for CD146 and PDGFRβ, supporting the idea that they are pericyte-derived tumours (Shen et al., 2015). The malignant forms show a decreased expression of pericytic markers suggesting that they become more undifferentiated cancer cells (Shen et al., 2015).

### 4.2 Pericyte density: role in tumour growth

Alterations in pericyte density appears particularly important as a prognostic marker in cancer (O'Keeffe et al., 2008, Cao et al., 2013, Xu et al., 2014). High pericyte coverage has been associated with forms of cancers that are the most aggressive and refractory to therapy (Furuhashi et al., 2004, Yonenaga et al., 2005).

**Fig. 2:**
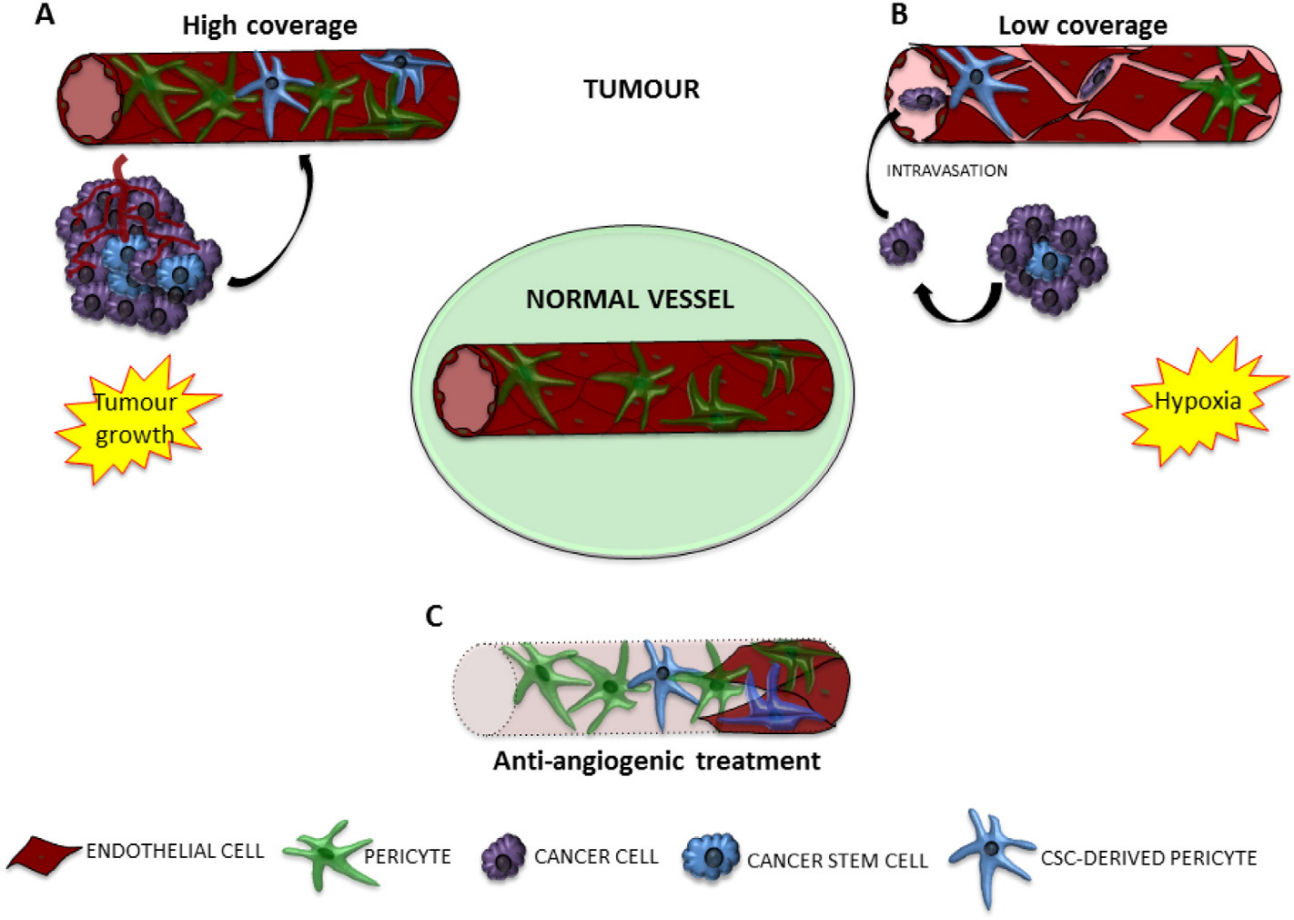
Interaction between pericytes and other vascular players in tumour. In normal condition ECs are stabilised by surrounding pericytes. In tumour this balanced is altered. A) In tumoural angiogenesis, pericytes could be recruited on site and stabilise the new formed vasculature *via* VEGF secretion. This can contribute to nourish the cancer cells, favouring tumour growth. Cancer cells can contribute to this process directly localizing in perivascular position. CSCs have the ability to differentiate in functional pericytes. B) Tumoural angiogenesis is highly disorganised, morphological changes do not allow cell-to-cell contact either between EC themselves or ECs and pericytes. The increase in vascular permeability facilitate the intravasion of cancer cells into the bloodstream. Hypoxia is a key condition for EMT to happen, promoting cancer cell mobilization. C) Drug resistance to anti-angiogenesis treatment can be due to pericytes. Therapy targeting ECs leave pericytes alive, forming a frame to be repopulated by ECs. Even in a combined strategy *versus* ECs and pericytes, CSCs in perivascular position can continue to act as pericytes.

In tumours, EC-mediated pericyte recruitment can be altered, resulting in different scenarios ranging from high to low pericyte coverage. As in the physiological state, PDGFb represents the main chemo-attractant for perivascular cells in cancer angiogenesis (Furuhashi et al., 2004, Suzuki et al., 2007). Other factors involved in pericyte recruitment are heparin-binding EGF-like growth factor (HB-EGF) (Nolan-Stevaux et al., 2010, Cascone et al., 2011), EC secreted SDF-1 (Song et al., 2009, Chen et al., 2012), and MMP-mediated ECM degradation (Chantrain et al., 2006). In addition, pericyte-derived VEGF and Ang-1 can nourish ECs, stabilising the vasculature and favouring tumour growth (Franco, Roswall, Cortez, Hanahan, and Pietras, 2011) (Fig. 2A).

Morphological changes, such as the discontinuity of endothelial basement membrane or multi-layered ECs, prevent pericytes making proper contact with ECs (Folkman, 2002, Baluk et al., 2003, Krishna Priya et al., 2016). Thus pericytes are forced away from the perivascular position, resulting in a leaky vessel (Glentis, Gurchenkov, and Matic Vignjevic, 2014) (Fig. 2B). Changes in the expression of adhesion molecules and secreted factors by pericytes can also contribute to tumourigenesis and tumour maintenance. In particular, the Notch pathway seems to be implicated in EC-pericyte interactions and stabilisation of the vasculature (Pedrosa et al., 2015). In the tumour microenvironment, reduced pericyte density leads to hypoxia, which triggers endothelial HIF-1α expression through a VEGF-mediated mechanism, promoting disorganised new vessel formation (Chen, Silva, Yuen, and Mooney, 2007).

Low pericyte density can also be associated with increased metastasis. This was elegantly demonstrated in a seminal paper from Xian et al., in which knocking out the adhesion molecule NCAM in a mouse tumour model was associated with increased metastasis (Xian et al., 2006). This observation has been confirmed in different tumours in which low pericyte coverage leads to increased tumour cell invasiveness, while a high coverage is correlated with a lowered rate of metastatic dissemination (Yonenaga et al., 2005, Ito et al., 2013, Jayasinghe et al., 2015, Zang et al., 2015, Correa et al., 2016).

Pericyte loss can also trigger epithelial-mesenchymal transition (EMT). This consists of the loss of epithelial features (cell polarity, basal lamina and adherens, tight, and gap junctions) followed by the acquisition of mesenchymal characteristics (spindle shape morphology, expression of fibronectin, vimentin, and N-cadherin) (Agrawal et al., 2014).

Moreover, high pericyte coverage could represent an obstacle for pericyte mimicry by CSCs. CSCs are a group of cancer cells characterized by the ability to self-renew and initiate tumour formation (Gupta et al., 2009, Welte et al., 2010). They also have the capacity to transdifferentiate to endothelial cells and pericytes, contributing to new vessel formation (Ricci-Vitiani et al., 2010, Wang et al., 2010, Cheng et al., 2013). Recent studies have demonstrated the ability of glioblastoma CSCs (GSCs) to give rise to a pericyte lineage either *in vitro* or *in vivo* (Scully et al., 2012, Cheng et al., 2013, Mao et al., 2013). Hence, GSCs are able to create a favourable TME which in turn facilitates tumour progression and dissemination. Not surprisingly, the most aggressive forms of glioma are characterized by pericyte hyperplasia (Xu et al., 2014).

### 4.3 Pericyte-mediated immunomodulation

Due to their close relationship with MSCs, which have immunomodulatory properties, it is not surprising that a growing body of evidence is emerging demonstrating how perivascular cells can also play a pivotal role in the recruitment and modulation of immune cells in the context of tumour formation.

Recent findings suggest that in tumours pericytes can act as immunomodulators of different leukocyte populations, with opposing results. Tumour-derived pericytes have the ability to negatively influence CD4^+^ lymphocyte proliferation and activation (Bose et al., 2013). In human malignant glioma, perivascular cell-like MSCs negatively correlated with CD8^+^ T cells, showing the ability to abrogate the CD8^+^ T cell response (Ochs et al., 2013). However, a xenotransplanted tumour in a pericyte-deficient mouse model showed a drastically increased recruitment of myeloid-derived suppressor cells (MDSC), an immature cell population inhibiting T-cell activation and proliferation. Restoring the normal pericyte coverage abrogated the increased MDSC trafficking in pericyte-deficient tumours (Hong et al., 2015). As mentioned above for tumour growth and metastasis, these findings demonstrate that pericytes can acquire different roles depending on their abundance and/or functionality.

## 5 Pericyte-targeted therapies: state-of-the-art and new frontiers

As discussed, pericytes exert a multitude of effects in many pathologies. However, as the importance of pericyte function in disease is a relatively recent discovery, only a few therapeutic strategies have specifically targeted pericytes.

A direct way to slow the progression of DR is to replace the pericytes lost in the early stages of NPDR. Adult MSCs isolated from the bone marrow can act as pericytes in certain conditions (Caplan, 2015). The RETICELL clinical trial is currently establishing whether an intravitreal injection of adult bone marrow MSCs to patients with retinitis pigmentosa can restore ocular function. This study has demonstrated that patients injected with MSCs have an increased quality of life up to 3 months after injection. Unfortunately, this effect is not detectable 12 months after treatment (Siqueira et al., 2015). In two studies of different mouse models of DN (T1D and T2D), injection of adult bone marrow MSCs have been shown to improve glomerular morphology, reduce mesangial thickening and limit macrophage invasion (Lee et al., 2006, Ezquer et al., 2009). These studies demonstrate that MSC/pericyte cell therapy to be a potentially efficacious method of curing diabetic nephropathy.

The angiogenic factor VEGF plays a major role in PDR and in tumour vasculogenesis, hence this factor has therefore been identified as a major target for therapeutics. The aim is to inhibit unrestrained endothelial sprouting and consequent uncontrolled neovascularisation. A current trend in DR therapeutic development is the generation of high-specificity, high-affinity antibodies targeting and inhibiting VEGF-A. Treatment with these antibodies has shown efficient restoration of ocular function in patients with DME (Wells et al., 2016). A long-term clinical trial testing the efficacy of the anti-VEGF Ranibizumab in patients with DME has also shown improved vision, sustained during the 5-year treatment programme (Elman et al., 2015).

Another strategy in DR is to limit the generation of ROS, which cause pericyte loss. PEDF is a secreted factor with potent antioxidant and anti-angiogenic capabilities mediated *via* the inhibition of VEGF expression (Yamagishi et al., 2002a, Zhang et al., 2006). A study using a solution of recombinant PEDF peptide applied topically to the eye of diabetic Akita mice showed promising results. The use of PEDF “eye drops” diminished pericyte cell death, vascular leakage, and inflammation in the retina (Liu et al., 2012b).

In diabetic nephropathies, pericyte-related therapies mainly focus on the inhibition of fibrosis. One of the protein targets for fibrosis abrogation is PDGF. PDGF is an important mediator of pericyte-myofibroblast transition and has been singled out as an important target in various fibrotic kidney diseases (Lin et al., 2008, Chen et al., 2011, Chang et al., 2012). Imatinib is a receptor tyrosine kinase inhibitor (RTK) specific for PDGFr and c-Abl. In an obese and diabetic mouse model, Imatinib protects against renal injury caused by the metabolic syndrome. Furthermore, Imatinib can also rescue the glomerular and tubule-interstitial structural damage caused by T2D as well as ameliorating albuminuria (Lassila et al., 2005).

In the case of tumour angiogenesis, an immense amount of resources have been dedicated to inhibiting or even removing the ECs at the heart of vasculogenesis. The most common type of treatment is, as with DR, anti-VEGF therapy, where the use of VEGF antibodies or antagonists for the VEGF receptor are administered to the patient. Anti-VEGF therapy is efficient acutely, but not chronically; inhibition of VEGF signaling can lead to 50–60% of tumour vasculature inhibition. However, after discontinuing treatment, the empty “vascular sheets” of collagen that used to host the vessels quickly become repopulated by new endothelial sprouts (Fig. 2C). This suggests that the undisturbed basement membrane, as well as the continued presence of pericytes in those vascular sheets, promote tumour re-vascularisation. Thus, it is now recognised that efficient therapies against tumour angiogenesis should target both ECs and pericytes (Mancuso et al., 2006).

PDFG receptor inhibitors such as Imatinib are also used as tumour anti-angiogenic therapies. Many clinical trials are currently evaluating the efficiency of anti-PDGF therapies on various cancers (reviewed in Heldin (2013)). For example, it has been shown that treatment of lymphomas with Imatinib inhibits angiogenesis. Imatinib appears to act on vascular mural cells which leads to the disruption of the integrity of the tumour blood vessels (Ruan et al., 2013). A study on the effect of mTOR inhibition with the drug Everolimus in conjunction with the PDGF receptor antagonist Nilotinib in gastric cancer has shown interesting results. While PDGFr inhibition did not lead to decreased tumour growth, and mTOR alone did not decrease tumour angiogenesis, a combination of the two caused decreased pericyte coverage, decreased stromal reactivity and decreased tumour growth (Onoyama et al., 2013).

However, anti VEGF-PDGF therapies are not a guaranteed success. Inhibition of VEGF and PDGF by bevacizumab and sunitinib respectively did not inhibit angiogenesis of glioma xenografts more effectively than VEGF inhibition alone (Navis et al., 2011). This failure of PDGF signaling inhibitors to enhance anti-VEGF therapy could mean that PDGF antagonism is not sufficient to disrupt pericyte function in the tumour vasculature. Recently, new targets have been identified and are gathering attention as possible therapies

One such novel anti-angiogenic target is Olfactomedin-like 3 (Olfml3), a member of the BMP family. Olfml3 is produced by both ECs and pericytes, is secreted in the perivascular region and has pro-angiogenic properties. Inhibition of Olfml3 in tumours has shown to affect both ECs and pericytes. Blockade of Olfml3 activity with specific antibodies has been shown to inhibit tumour vascularization, pericyte coverage and tumour growth in a mouse xenograft cancer model (Miljkovic-Licina et al., 2012).

Another strategy being evaluated is the direct disruption of endothelium-pericyte interactions to destabilise the vasculature. Endosialin/CD248 is expressed in tumour-associated pericytes (Valdez, Maia, and Conway, 2012). Application of the CD248-specific antibody MORab-004 in a mouse xenograft model caused a decrease in primary tumour growth. Analysis of the tumour vasculature showed an increase in smaller and non-functional microvessels. MORab-004 treatment induces internalization of CD248 by pericytes, which coincides with a depolarization of both pericytes and ECs, suggesting that CD248 acts as a scaffold or guide for tumour microvasculature (Rybinski et al., 2015).

Another elegant strategy involving pericytes does not try to destroy cancer pericytes *per se*, but use their specific properties to target tumours. An interesting new concept in the field of anti-angiogenic tumour treatment is to induce a so-called “tumour infarction”, where the tumour vasculature is blocked. These infarcts are induced by administering a truncated version of Tissue Factor (tTF), which has the ability to induce thrombosis (Huang et al., 1997). Brand et al. have used both the leaky properties of tumour endothelium and specific pericyte markers to target tTF to tumour sites. Pro-angiogenic cancer pericytes express the surface proteoglycan NG2. Treatment of tumour-grafted animals with a tTF-NG2 antibody fusion protein have shown that tTF can be delivered to the tumour vasculature, can activate coagulation within the microvasculature and inhibit tumour growth (Brand et al., 2016). Guan et al. have also used NG2 to target an anti-tumoural drug to the tumour site. They have developed a system by which a nanoparticle conjugated to a peptide recognising a NG2 epitope can be directed to the tumour site. This group has shown that their TH10-nanoparticle containing the anti-mitotic drug docetaxel targets cancer pericytes and is then internalized. This internalization causes cancer pericyte cell death and a marked decrease in tumour angiogenesis (Guan et al., 2014).

Counterintuitively, certain therapeutic targets increase pericyte coverage to normalize tumour vasculature. Inhibition of either TRPV4 or Ang2 in mouse Lewing lung carcinoma or mouse glioblastoma *multiforme* respectively decreased tumour growth while increasing pericyte coverage (Adapala et al., 2016, Scholz et al., 2016, Thoppil et al., 2016). Increased pericyte coverage of the tumour vasculature decreases the permeability of the vessel thus limiting the access of the tumour to pro-inflammatory and pro-tumourigenic cells.

Another important player in tumour vasculogenesis are CSCs, which can take on the role of pericytes and contribute to the formation of new vessels. As previously mentioned, the Notch signaling pathway is an important regulator of CSCs. G-secretase inhibitor (GSIs) can be used to inhibit the Notch membrane proteins, thus blocking Notch signaling. Treatment of glioblastoma tumours with GSIs led to the depletion of stem-like cells in the tumour and to a decrease in tumour growth (Fan et al., 2010).

In the last ten years, microRNAs have been gathering more attention in the field of cancer therapeutics. While endothelial and angiogenesis-related microRNAs are well characterized, very little is known about pericyte-specific microRNAs (Kuninty, Schnittert, Storm, and Prakash, 2016). It would be interesting to see in the future if the selective disruption or activation of certain microRNAs could contribute to the inhibition of tumour angiogenesis.

### 5.1 The blood brain barrier, pericytes and drug delivery to the CNS

The BBB is a specialised structure composed of ECs, astrocytes, microglial cells, neuronal projections and pericytes. Previously it has been shown that the molecules diffusing through the BBB appear to be lipophilic, smaller than 600 Da in size, with or without a specific affinity for endogenous cell transport receptors (Pardridge, 2007).

Pericytes are very important to support cells for this endothelial barrier and are essential for competent BBB function (Daneman, Zhou, Kebede, and Barres, 2010). In a pericyte-deficient mouse model, it has been shown that pericytes contribute to the BBB by regulating gene expression patterns in ECs and by inducing polarization of astrocyte end-feet surrounding CNS blood vessels (Armulik et al., 2010). It has also been shown, in an *in vitro* co-culture model of BBB, that pericytes are essential for BBB function. Indeed, the addition of pericytes to an EC monolayer increased transendothelial resistance and decreased lipophilic molecule permeability (He, Yao, Tsirka, and Cao, 2014b), partly by increasing the expression of the protein occludin, the main contributor to the tight-junction organisation (Dohgu et al., 2005). Interestingly, pericytes can themselves actively participate in allowing both molecules and cells through the BBB. The pericytes can act as an “enzymatic barrier” by secreting different peptidases that can break down protein and high-molecular weight peptides, preventing them from crossing to the brain (Omidi and Barar, 2012). Moreover, pericytes are involved in the recruitment of neutrophils to the sub-endothelial layer and the breaching of the vascular wall by secreting the chemoattractant IL8 and the metalloproteinases MMP-2 and MMP-9, which break down endothelial cell-to-cell contacts (Proebstl et al., 2012, Pieper et al., 2013). Pericytes can also play a role similar to macrophages. They express scavenger receptors that bind various antigen-bound antibodies. This activates phagocytosis, allowing the pericytes to clear cellular debris and parasites (Jansson et al., 2014).

As previously mentioned, the BBB is a major barrier to the many molecules that could negatively affect the CNS. Unfortunately, this exquisitely regulated system is a hindrance when pharmaceutical compounds need to be delivered to the CNS. Currently, close to 98% of small molecule and 100% of large molecule drugs on the market do not enter the brain or cannot achieve concentrations needed for therapeutic benefits (Pardridge, 2007). Current strategies to deliver drugs to the brain include improved lipophilicity of the compound, lipid/vesicle packaging of the active agent, targeting specific transporters by linking to a receptor ligand, “trojan horse” mimic or direct disruption of the BBB. Most of these strategies focus on the endothelial barrier itself, and very few studies target the other cell types forming the BBB (Banks, 2016). It is plausible that a shift in focus to the cells regulating the endothelial barrier could have substantial benefits on future strategies in drug design. One could take advantage of the increased trans-endothelial “leakiness” encountered when BBB pericytes are receiving inflammatory signals (Banks, 2016). Conjugation of drugs to antibodies could capitalise on the macrophage-like activity of the BBB pericytes by activating phagocytosis, thus bringing the active compound further through the barrier (Banks, 2016).

## 6 Concluding remarks

In the last twenty years, our knowledge of pericytes has evolved considerably. Pericytes have been better characterized and isolated from several tissues, revealing universal and tissue-specific functions. Moreover, pericytes have been shown to have a multipotent lineage ability, similar to other perivascular populations such as MSCs and adventitial progenitor cells, with which they share common markers and properties. These heterogeneous populations could be grouped as PSCs. This characteristic favours the usage of PSCs in the field of regenerative medicine and tissue engineering. However, it is still not completely clear how PSCs are related among themselves and which one could be the most appropriate for cell therapies. The different tissues of origin, the differences in functions and phenotype and the lack of standardization in the clinical trials so far call for an urgent basic science quest to resolve these concerns. There is a risk of repeating the same mistakes seen with vascular progenitor populations isolated from peripheral blood or bone marrow. The lack of scientific consensus on characterization and standardization in isolation protocols, use in *in vivo* animal model and in clinical trials has led to a large amount of data that is challenging to analyse collectively (Ikebe and Suzuki, 2014, Ullah et al., 2015).

As per phenotypic and functional characterization, the knowledge of pericyte involvement in pathobiological events has grown exponentially in recent years. If the succession of pathological events, well-established in diseases such as diabetes, is observed in other diseases, new pathobiological scenarios will appear. For instance, the ability of pericytes to differentiate into fibroblast cells supports their involvement in post-ischemic fibrosis (Birbrair et al., 2014). The stem cell-like nature of pericytes has emerged in their relationship with CSCs, along with their immunomodulatory properties, providing new explanations for pharmacological resistance (Ullah et al., 2015).

In light of these discoveries, new specific therapeutic approaches have been developed. The affinity of pericytes with MSCs has led to the use of the latter in clinical trials in patients who suffer from retinopathy. In tumours, pericytes, along with ECs, have become a major target of anti-angiogenic therapies. Moreover, the discovery of particular signaling pathways, such as the Notch-related pathway, allows the development of novel approaches that can be used in a multi-strategic co-adjuvant cancer therapy. In addition, diseases such as diabetes can provide an epigenetic modification to PSCs, which may be a key factor in cell therapy based regenerative medicine treatment (Gubernator et al., 2015).

For all these reasons, we would like to underline the importance of three pivotal fields that should be thoroughly investigated in pericyte biology in the future:

a) Characterization of pericyte sets and subsets, embryological origin, and location in the hierarchy of stem and progenitor cells in adult tissues.
b) Novel signaling pathways affected in pathological events, which could represent novel targets for disease treatments.
c) Standardization of methods for the use of pericytes in clinical trials.

Accordingly, pericytes, for a long time an overlooked player in pathobiology, could become a candidate for better tailored therapeutic treatment, as well as a powerful tool for tissue engineering and regeneration.

## Conflict of interest statement

The authors declare that there are no conflicts of interest.

## Funding acknowledgements

DF, JR and GM are supported by the British Heart Foundation “Unravelling mechanisms of stem cell depletion for preservation of regenerative fitness in patients with diabetes” programme grant (RG/13/17/30545). SS is supported by the Heart Research UK “Unravelling the molecular mechanisms of human adventitial pericytes for clinical translation” project grant (UK-RG2639). This article received also the support of the British Heart Foundation Research Centres for Excellence network (RM/13/2/30158).
